# Weighting the factors affecting attention guidance during free viewing and visual search: The unexpected role of object recognition uncertainty

**DOI:** 10.1167/jov.22.4.13

**Published:** 2022-03-24

**Authors:** Souradeep Chakraborty, Dimitris Samaras, Gregory J. Zelinsky

**Affiliations:** 1 Department of Computer Science, Stony Brook University, Stony Brook, NY, USA; 2 Department of Computer Science, Stony Brook University, Stony Brook, NY, USA; 3 Departments of Psychology and Computer Science, Stony Brook University, Stony Brook, NY, USA; 4 Department of Psychology, Stony Brook University, Stony Brook, NY, USA; 5 Department of Computer Science, Stony Brook University, Stony Brook, NY, USA

**Keywords:** uncertainty, attention, fixations, saliency, visual search

## Abstract

The factors determining how attention is allocated during visual tasks have been studied for decades, but few studies have attempted to model the weighting of several of these factors within and across tasks to better understand their relative contributions. Here we consider the roles of saliency, center bias, target features, and object recognition uncertainty in predicting the first nine changes in fixation made during free viewing and visual search tasks in the OSIE and COCO-Search18 datasets, respectively. We focus on the latter-most and least familiar of these factors by proposing a new method of quantifying uncertainty in an image, one based on object recognition. We hypothesize that the greater the number of object categories competing for an object proposal, the greater the uncertainty of how that object should be recognized and, hence, the greater the need for attention to resolve this uncertainty. As expected, we found that target features best predicted target-present search, with their dominance obscuring the use of other features. Unexpectedly, we found that target features were only weakly used during target-absent search. We also found that object recognition uncertainty outperformed an unsupervised saliency model in predicting free-viewing fixations, although saliency was slightly more predictive of search. We conclude that uncertainty in object recognition, a measure that is image computable and highly interpretable, is better than bottom–up saliency in predicting attention during free viewing.

## Introduction

People shift their attention as they perform different visual tasks, with overtly measurable changes in eye fixation numbering in the hundreds of thousands each day. There is a sizeable literature aimed at identifying factors affecting these ubiquitous movements of attention. Two broad factors that are well known to guide attention are target goals, as studied by visual search tasks, and bottom–up visual salience, as studied in the context of free-viewing tasks. Less studied factors have also been identified, ranging from simple center biases to uncertainty in the recognition of a scene's objects. The relative weighting of saliency and target features in a visual search task has been investigated (Chen & Zelinsky, 2006; Nothdurft, 2006; Zelinsky, Zhang, Yu, Chen, & Samaras, 2006; Ehinger, Hidalgo-Sotelo, Torralba, & Oliva, 2009; Henderson, Malcolm, & Schandl, 2009; Lamy & Zoaris, 2009), where the target features have been determined to be significantly more important than saliency features in visual search. Several other previous works have also studied the relative importance of some of these factors for free viewing (Mancas, 2009; Hayes & Henderson, 2019; Schütt, Rothkegel, Trukenbrod, Engbert, & Wichmann, 2019). However, the relative importance of all of these factors has not been considered in the context of a single study, and this is the focus of our work.

We briefly review the visual search and/or free viewing studies that have considered each of four factors (saliency, target features, center bias, and uncertainty), but we limit our discussion to studies that used computationally mature methods that can be applied to natural images. We do this to approximate the visually complex contexts in which these shifts of attention naturally occur and to promote fair model comparison. We also present an extended discussion of uncertainty, given that the study of this factor in guiding attention has been relatively neglected compared with the roles of saliency and target features. We have formulated a new measure for quantifying the uncertainty in the recognition of an object and present a framework for combining this factor with the others to show how each was differentially weighted across the first nine fixations in both free-viewing and search datasets. We end with some interpretation of what the observed weightings might suggest, focusing again on the role of object recognition uncertainty in guiding overt visual attention.

### Bottom–up saliency

There are several recent reviews detailing the relationship between image saliency and the attraction of attention, as well as large datasets of free-viewing behavior that have been created to train and test models of saliency (Judd, Ehinger, Durand, & Torralba, 2009a; Xu, Jiang, Wang, Kankanhalli, & Zhao, 2014; Jiang, Huang, Duan, & Zhao, 2015). Indeed, saliency models that predict free-viewing fixations have become such an active research topic that a managed benchmark has emerged to compare all of the models on a level playing field (Bylinskii, Judd, Borji, Itti, Durand, Oliva, & Torralba, 2015). Rather than duplicating these reviews, here our focus is on clarifying how we define saliency in our study and the related concept of priority. Different definitions of saliency produce different levels of success in model predictions of free-viewing fixations. The formulation of saliency that we use is one proposed by Harel, Koch, and Perona (2007). Details are provided in the Methods section, but it builds on the method proposed by Itti and colleagues (Itti, Koch, & Niebur, 1998; Itti & Koch, 2001) that computes saliency in terms of local feature contrast in intensity, color, and orientation in a visual input. This method is one of the best in a class of methods that define saliency purely as a bottom–up visual process, such that the computation of the saliency map uses no knowledge other than what is contained in the pixel input. We use the term “saliency model” to refer to one that relies on no top–down input, including any object categories that were learned during training and can now serve as a top–down bias signal.

A purely bottom–up saliency model can be contrasted with a model that combines bottom–up pixel input with top–down biases (e.g., faces, text, target-object goals). We refer to these methods as computing “priority maps,” a different term to underscore the critical difference from bottom–up saliency in their use of top–down information in the attention predictions. Many types of top–down biases exist, and we confine our discussion to only one of these—the biasing of attention to target features in a search task. For even greater specificity, we refer to priority maps in the context of a search task as “target maps” (Zelinsky, 2008), a name that makes clear that the prioritization is based on a comparison of a visual input to features of a target goal. We adopted and consistently used this terminology in several recent studies (Yang et al., 2020; Zelinsky et al., 2020b), most clearly defined in Zelinsky and Bisley (2015), and we believe that these distinctions are particularly useful given our present goal of better understanding how different attention biases are weighted in the context of free-viewing and search tasks. In our view, when an assumption of top–down input is made, even in cases of simple text and face detection (Liao, Shi, Bai, Wang, & Liu, 2017; Boyko, Basystiuk, & Shakhovska, 2018; Long, Ruan, Zhang, He, Wu, & Yao, 2018), a mixture of priority signals occurs that makes it challenging to compare models, with model performance often correlating with how much top–down input can be added to the prediction. Such mixing can be useful if the goal is to best predict fixation behavior, but this was not our goal in this study.

### Target features

Eclipsing in size even the robust literature on saliency, the use of top–down goals and target features to guide attention has been studied for decades in the context of visual search (Horowitz & Wolfe, 1998; Weidner, Krummenacher, Reimann, Müller, & Fink, 2009; Wolfe & Horowitz, 2017). Overt movements of attention are biased to the features of a target, so much so that a target category can be decoded from the eye movements made even during target-absent (TA) search (Zelinsky, Peng, & Samaras, 2013). Moreover, this top–down bias is known to be different from the bottom–up biasing of attention captured by models of saliency (Chen & Zelinsky, 2006; Henderson, Brockmole, Castelhano, & Mack, 2007; Koehler, Guo, Zhang, & Eckstein, 2014), meaning that saliency model predictions do not generalize to search tasks. Most theories of visual search explain target guidance as a comparison process between a representation of a target goal and a visual input (Duncan & Humphreys, 1989; Wolfe, Cave, & Franzel, 1989; Treisman, 1991). The hugely influential *guided search model* made this comparison explicit in the context of several simple search tasks and patterns of button-press responses (Wolfe, 1994), and the *target acquisition model* later extended this computational approach by using target maps to predict search fixations on complex images (Zelinsky, 2008).

Although early models of search guidance used target features that were known to the searcher, an advance in search theory came with the demonstration that search can be guided even to targets defined by an object category (Malcolm & Henderson, 2009; Schmidt & Zelinsky, 2009; Yang & Zelinsky, 2009). Soon afterwards, models began to generate categorical target maps to predict the fixation made in the search for common object categories (Zelinsky, Adeli, Peng, & Samaras, 2013; Adeli, Vitu, & Zelinsky, 2017; see Zelinsky, Chen, Ahn, & Adeli, 2020a, for a recent review of models of search-fixation prediction). This extension of search theory to object classification was significant in the bridge that it built to computer vision, where powerful methods have been developed for extracting and detecting objects in images by learning robust object category representations. Using this bridge, the recent models that predict search fixations are all deep networks (Wei, Adeli, Nguyen, Zelinsky, & Samaras, 2016; Adeli & Zelinsky, 2018; Zhang, Feng, Ma, Lim, Zhao, & Kreiman, 2018), with the current state of the art being a model that predicts search fixations using a prioritization policy learned through imitation of previously observed search behavior (Yang et al., 2020). The dataset of search fixations that we use in the present study, COCO-Search18, was developed to provide the observations of search behavior needed by this model for training (Chen et al., 2020). However, it is not our current goal to set a new benchmark by outperforming these models or even to enter into the arena of search-fixation prediction. Rather, here we use a simpler modeling framework (He, Gkioxari, Dollar, & Girshick, 2020), yet one still complex enough to prioritize categories of objects in real- world images, to obtain object and target maps that can be compared with the other biases considered in our study.

### Center bias

Center bias refers to a tendency to allocate attention preferentially toward the center of an image. Part of the center bias can be explained by the fact that much of the imagery that we consume daily was created by people who deliberately framed the image to place an object of central interest at the center (Tatler, 2007; Marat, Rahman, Pellerin, Guyader, & Houzet, 2013). Consequently, viewers learn that the center of an image should be biased for attention priority. Center bias is a significant predictor of eye position in arbitrary natural scenes, with simple center bias models even outperforming more complex models that do not include a center bias (Le Meur, Le Callet, & Barba, 2007; Judd, Ehinger, Durand, & Torralba, 2009b).

However, the center bias is likely itself a mixture of many weak biases. Upon first viewing a scene people tend to direct their initial saccades toward locations closer to the center (Renninger, Verghese, & Coughlan, 2007; Tatler, 2007; Zelinsky, 2012), with further scene exploration then commencing from this center location. A center bias might therefore be functional in conveying an information processing advantage by establishing an optimal starting position for exploring a scene with a foveated retina. In addition to such strategic factors, center bias might also include low-level motor factors used to re-center the eye in its orbit and higher level biases stemming from blurred peripheral information competing less successfully for attention than less blurred information in the nearer periphery (Tseng, Carmi, Cameron, Munoz, & Itti, 2009; Zhao & Koch, 2011). Important for our study, center fixation bias has also been shown to persist irrespective of the distribution of image features, or the observer's task. This suggests a relatively simple image- and task-independent bias to allocate attention to the center of a scene (Tatler, 2007), one largely divorced from the features of the image, and it is this simple definition of a center bias that we adopt in our work.

### Uncertainty of object recognition

The idea that attention is biased to regions in an image having uncertain content dates back to Renninger, Coughlan, Verghese, and Malik (2005), who proposed that fixation selection during scene viewing follows a principle of uncertainty minimization. At about the same time, a different perspective on uncertainty was proposed by Itti and Baldi (2006), who defined uncertainty as a mismatch between prior and posterior model probabilities, with greater mismatch corresponding to higher uncertainty. In a later extension of this work, Baldi and Itti (2010) introduced a model of Bayesian surprise and formulated its relationship to Shannon entropy. In another influential and complementary study, Bruce and Tsotsos (2009) also conceptualized uncertainty as surprise, but one that is localized to a region based on a principle that they referred to as *self-information*. If the content of an image region can only be poorly predicted by the surrounding contextual information in the image, then there is higher uncertainty there and the greater potential for surprise when attention moves to that region. In more statistically oriented approaches, Feldman and Friston (2010) argued that free energy and the Fisher information of an image are useful measures of the uncertainty associated with an image region, and Sullivan, Johnson, Rothkopf, Ballard, and Hayhoe (2012) treated uncertainty as the variance of the probability distribution associated with a belief that the world is in a particular state given a set of visual observations over time. Relatedly, Standvoss, Quax, and Van Gerven (2020) suggested that uncertainty can be characterized by the variability in how well an unsupervised method (a variational autoencoder) can reconstruct an image, where they defined uncertainty at each image location based on the variability among five reconstruction samples. What these studies have in common is the belief that attention is allocated to maximize a sort of surprise by minimizing uncertainty. Here, we build on the idea that attention allocation is prioritized to minimize uncertainty but extend it by proposing an uncertainty metric more focused on object recognition.

Our focus on objects is motivated by the large literature suggesting that high-level vision is biased to perceive objects and that objects are the unit of selection by spatial attention (Mozer & Sitton, 1998; Scholl, 2001; Walther & Koch, 2006). Applying object-based attention to fixation prediction, Einhaüser, Spain, and Perona (2008) and Stoll, Thrun, Nuthmann, and Einhaüser (2015) found that the object locations in an image predicted where people fixate better than low-level salience, and they showed this to be true in artistic evaluation, content analysis, object naming, and visual search tasks. Several other studies highlight the importance of objects in scenes by incorporating object representations into attention-prediction models ('t Hart, Schmidt, Roth, & Einhaüser, 2013). Chang, Liu, Chen, and Lai (2011) proposed a computational exploration of the relationship between objectness and saliency, and Ji, Zhang, Tseng, Chow, and Wu (2019) considered both an objectness cue and saliency detection in a graph-based bottom–up salient object detection framework. Object representations are also explicitly or implicitly assumed by many studies using deep neural network models (Kümmerer, Wallis, & Bethge, 2016; He, Tavakoli, Borji, Mi, & Pugeault, 2019; He et al., 2020), given that these networks are often pretrained on large datasets that where labeled for object classification. For example, DeepGaze II (Kümmerer et al., 2016) is a model that predicts free-viewing fixations using the features of a VGG-19 deep neural network that was trained on ImageNet (Deng, Dong, Socher, Li, Li, & Fei-Fei, 2009) to identify objects in images. Also relevant is a study by Chen and Zelinsky (2017, 2019), where free-viewing fixations were predicted using a combination of saliency and mid-level representations of shape referred to as proto-objects. They showed that their model better predicted free-viewing fixations than a bottom–up saliency model, which they interpreted as attention selecting object-like regions of space. These same authors (Chen & Zelinsky, 2018) challenged the assumption that free-viewing fixations reflect bottom–up salience. They did this by introducing an object-based model that used top–down biases from learned object representations obtained using a state-of-the-art convolutional neural network pre-trained for object classification (using 1000 object categories from ImageNet) (Krizhevsky, Sutskever, & Hinton, 2012) to predict the fixations made during free viewing. They hypothesized that the same visual object representations learned and used to control goal-directed search behavior might also be collectively engaged to guide attention more generally to objects (rather than targets) in the absence of a goal. Because multiple object representations might simultaneously exert a guidance signal, the contribution of any one is diluted and a more generalized object-guidance signal is obtained. Supporting their hypothesis, these authors found that this object-based model outperformed bottom–up saliency models in predicting free-viewing fixations.

Building on this work, here we suggest that a factor affecting attention guidance may be uncertainty in the object-category label that should be assigned to a given object in the visual input (from among those object categories that have been learned). For intuition, if the features at one peripherally viewed location of a scene activate representations for a fork, knife, and spoon, whereas the features at another location are plausibly recognized as either a plate or a bowl, the former pattern arguably has greater object uncertainty than the latter given that viewing state. We treat this object recognition uncertainty as a priority signal and use it to predict free-viewing and search fixations.

## Methods

Many factors are known to affect the allocation of attention during free-viewing and visual search tasks, and here we provide details regarding the four factors that we consider in this study—namely, object recognition uncertainty, center bias, bottom–up saliency, and guidance from target features in the case of search.

### Object recognition uncertainty

It is possible to know that there is an object in an image without knowing what that object is, particularly for objects that have not yet been fixated. To make this phenomenology computationally explicit, for each yet-to-be- recognized *object proposal* in an image we estimate the number of learned object categories that compete for classification of that object-proposal bounding box. We then compute from these competing categories an object uncertainty score, where a larger number of categories competing for an object proposal reflects greater uncertainty in how that object should be recognized. To obtain object proposals and object detections, we used MaskRCNN (He et al., 2020), an instance segmentation method popular in the computer vision literature. We formulate the proposed uncertainty measure as follows:

(1) $$\begin{array}{c} UC=\sum_{b=1}^{B}G_{B_{center}}^{\sigma }\left(\frac{f_{b}}{max_{B}f_{b}}\right)\; \end{array}$$

where *B* is the number of object proposals (bounding boxes) in image *I* obtained using non-maximum suppression (with value, *Th_NMS_*) on the object proposals generated using the MaskRCNN method for instance segmentation; *f_b_* is the number of competing objects for a proposal *b*; and $G_{B_{center}}^{\sigma }$ is a Gaussian kernel, with *G* centered at the proposal box center with standard deviation (*SD*) σ. Here, *max_B_f_b_* indicates the maximum value that *f_b_* obtains across all object proposal bounding boxes (*B*) in image *I*.

**Figure 1.**
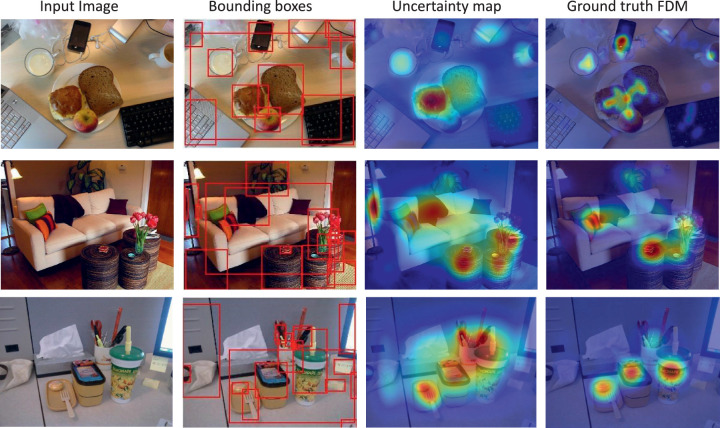
Examples of MaskRCNN-generated object proposal bounding boxes, shown with the corresponding uncertainty maps and the ground-truth fixation-density maps.

Equation 1 is used to compute an uncertainty (UC) priority map. Specifically, we first computed uncertainty values for every object proposal bounding box in the image, as described above. Because these bounding boxes can overlap extensively in an image, we used non-maximum suppression to eliminate object proposal boxes having high spatial overlap for the same object instance. This creates a one-to-one mapping between an object proposal and a unique object in an image. However, object proposals from different objects can also overlap. To deal with this eventuality, we define the uncertainty value at pixel *P* to be the summed uncertainty arising from all of the overlapping bounding boxes that include pixel *P*. Figure 1 visualizes the object bounding boxes generated using MaskRCNN along with the corresponding uncertainty priority map. We used an object detection confidence threshold, *Th_conf._* = 0*.*02, and a non-maximum suppression threshold, *Th_NMS_* = 0*.*30, for all analyses using the UC model in this study. Our uncertainty prediction algorithm can be summarized as follows:

**Table:** 

**Algorithm 1. Our uncertainty prediction algorithm**
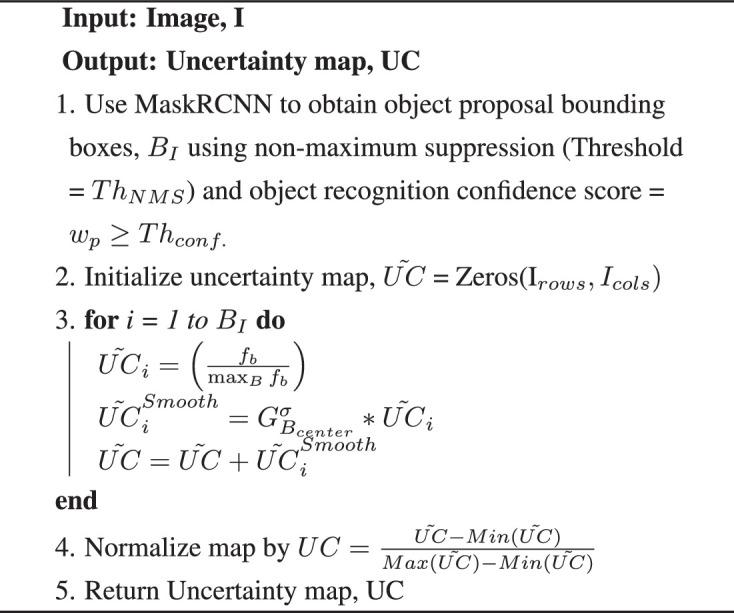

Our proposed measure of object recognition uncertainty can also be interpreted from an information theoretic perspective. We define, *P_unique_*(*B_I_*) to be the probability that an object in proposal bounding box *B_I_* is uniquely recognized as an object, which is equivalent to the probability that *f_b_* = 1 in Equation 1. The associated self-information can then be defined as *SI_unique_*(*B_I_*) = –log *P_unique_*(*B_I_*). To make this clearer, consider four proposal boxes, A, B, C, and D, in an image with the number of competing objects for each being 3, 2, 1, and 4, respectively. The corresponding probabilities of these boxes containing a single unique object are: $1-\frac{3}{\left(3+2+1+4\right)}=0.70,\;1-0.2=0.80,\;1-0.1=0.90,\;1-0.4=0.60$, respectively, after normalization. The self-information (*SI_unique_*) associated with boxes A, B, C, and D would therefore be *SI_unique_*(A) = 0*.*154, *SI_unique_*(B) = 0*.*096, *SI_unique_*(C) = 0*.*045, and *SI_unique_*(D) = 0*.*221, respectively. Therefore, box D has the highest self-information, *SI_unique_*(D), as *P_unique_*(D) has the lowest probability, *P_unique_*(D) = 0*.*6, of containing a unique object, making object proposal box D having the highest associated uncertainty. In this sense, our method of quantifying uncertainty with respect to object recognition can be understood as a maximization of self-information associated with object proposals.

### Bottom–up saliency

For a model of bottom–up saliency (Sal) we used graph-based visual saliency (GBVS) (Harel et al., 2007). Like other formulations of bottom–up saliency, it seeks to emulate the center-surround mechanism used by early visual areas to code contrast in a visual input, under the assumption that greater feature contrast leads to a greater probability of attracting attention. We used the MATLAB implementation of GBVS (Koch, 2012), which computes feature maps using the Itti–Koch saliency method (Itti et al., 1998) but normalizes these maps using a graph-based approach to highlight the conspicuous image regions and to permit combination with other importance maps. Specifically, a graph having feature vectors computed on image regions as its nodes is constructed, and a Markov chain is defined by normalizing the weights of the outbound node edges to 1. The equilibrium distribution of this chain reflects the fraction of time a random walker would spend at each node/state if it were to walk forever. This distribution naturally accumulates mass at nodes having high dissimilarity with their surrounding nodes because transitions among nodes corresponding to dissimilar image features is more likely than among nodes with similar features. This creates an activation measure that is derived from pairwise contrast and results in a biologically plausible model. As already discussed, although more recent methods are more predictive of free-viewing fixation locations than the GBVS model, these methods blur the distinction between bottom–up saliency and learned object categories by training on class labels, thus confounding our effort to tease apart the underlying factors affecting overt attention in our tasks. Among the other purely bottom–up saliency methods, there is adaptive whitening saliency (AWS) (Garcia-Diaz, Leboran, Fdez-Vidal, & Pardo, 2012) and Signature (Hou, Harel, & Koch, 2011), but we found in pilot work that all of these recent saliency models yield similar patterns of results that would not affect any of our main conclusions.

### Center bias

We implemented a center bias model (*CB*) by computing a two-dimensional (2D) Gaussian map centered on image *I_c_*(*x*_0_*, y*_0_), with its size determined by the image dimensions. More specifically,

(2) $$\begin{array}{c} CB_{p}=\frac{1}{\sigma_{c}\sqrt{2\pi }}e^{-{\left(P-I_{c}\right)}^{2}/2\sigma_{c}^{2}}\; \end{array}$$

where *CB_p_* denotes the Gaussian map value at image pixel *P*, and *σ_c_* is the standard deviation of the 2D Gaussian function. This center bias formulation is similar to what was used in previous studies (Marat et al., 2013; Tong, Lu, Zhang, & Ruan, 2014).

### Target-object features

Target-object guidance is believed to result when image locations have features similar to those of the target-object representation (Zelinsky et al., 2020a). Target guidance is therefore strongest on target-present (TP) trials where a target actually appears in the image, but a similarly computed, albeit weaker target guidance exists in TA search. To study how target guidance in a search task compares to center bias, saliency, and object recognition uncertainty, we need a method for obtaining a target map that reflects a bias for target features in a visual input. As already reviewed, there are many methods for doing this, but in the interest of keeping the state representations as comparable as possible in our model comparison we used the same MaskRCNN object proposal method (He et al., 2017) that we used to obtain an object uncertainty map. However, different thresholds on confidence were used depending on whether the search was target present or target absent. For TP search, we obtained the MaskRCNN object proposal bounding box in the image that had a confidence score greater than 0.9 that the object was an exemplar of the target category. We chose this high confidence threshold to ensure that the target was the only object selected in the scene, which was true most of the time. Moreover, the intersection over the union of this bounding box with the ground truth target-object labels from COCO-Search18 was 0.826, thereby validating our use of the MaskRCNN method. We then obtained a target map (Target) by applying a 2D Gaussian (*σ* = one-fourth of the box height, *h_b_*, as done for the center bias map, and size = image height, *h_im_*, resized to the box dimensions) at the center of this bounding box. In the case of TA search, we simply lowered the level of confidence for the MaskRCNN to 0.02, which was necessary because the confidence of a non-target object being the target is usually much lower compared with the confidence of actual target objects. A target map was then obtained similar to TP search. Specifically, we applied the same 2D Gaussian used for the TP search at the center of every bounding box (with recognition confidence value *>* 0*.*02) to obtain the target map, again assuming that there are some features at the bounding box locations that are guiding attention in proportion to their target similarity. Note that, whereas more sophisticated methods have been developed for predicting search fixations (Yang et al., 2020; Zelinsky et al., 2020a), we thought it best to err on the side of interpretability when selecting a method for obtaining a target map, which is often a problem for more sophisticated deep-learning methods. Our implementation of a target map is a simple bias much like a center bias, only the bias is introduced at the detected target locations. Given our goal of weighting the contributions of different features in a comparison, and not best predicting fixation locations, we believe this interpretability of the MaskRCNN method is a strength.

### Fixation datasets

We used two benchmarked and publicly available datasets of fixation behavior, one collected during a free-viewing task and the other during a visual search task. The OSIE dataset (Xu et al., 2014) consists of 700 images that were segmented into 5551 contoured objects, each rated for 12 semantic attributes (e.g., color, motion, watchability). Critically for our purpose, these images were also annotated with the fixations of 15 people freely viewing each of the depicted scenes for 3 seconds. We excluded from this dataset images that contained humans or animals in order to avoid known biases to these categories that strongly attract attention but are not among the biases that we consider in this study. After filtering out these object categories, which we did by using the corresponding MaskRCNN channels to detect these categories in the images, we were left with 145 images for analysis. Surprisingly few datasets have been developed for visual search behavior, but by far the largest is COCO-Search18 (Chen, Yang, Ahn, Samaras, Hoai, & Zelinsky, 2021; Yang et al., 2020). It consists of roughly 300,000 fixations from 10 people searching for each of 18 target-object categories in 6202 images of natural scenes. Participants made a TP or TA search decision for each image, and data were grouped to obtain 3110 TP images and 3108 TA images for analysis.

## Results

**Figure 2.**
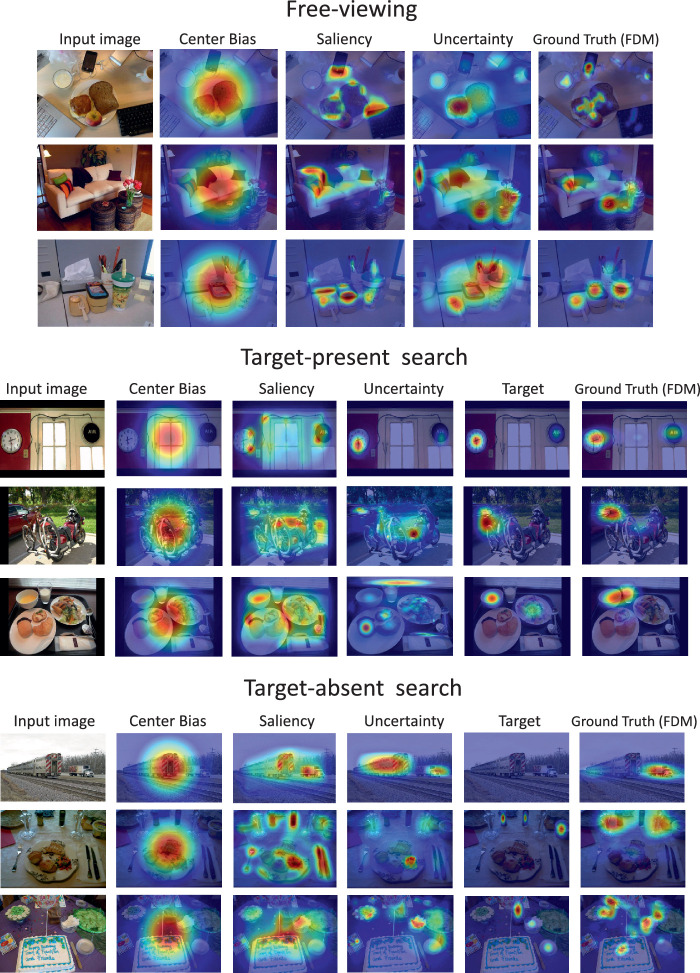
The four priority maps (center bias, saliency, uncertainty, and target) shown with the original input image (leftmost) and the ground-truth fixation-density map (rightmost) for three representative trials in free viewing (top), TP search (middle, where the target objects are a clock, car, and bowl in rows 1–3, respectively), and TA search (bottom, where the target objects are a car, bottle, and cup in rows 1–3, respectively).

To obtain a broad view of the results, in Figure 2 we show visualizations of how center-bias maps, saliency maps, uncertainty maps, and target maps predict ground-truth fixation-density maps (FDMs) for several representative input images. The three main panels of the figure show data from three different tasks: free viewing (top), TP search (middle), and TA search (bottom). Note that priority in the uncertainty maps is more focused on objects than in the corresponding saliency maps and therefore better approximates the free-viewing FDMs. Note also that this object bias persisted in the context of a search task, although now the target map does the best job in predicting fixation behavior. This superiority of a target map was expected in the context of TP search, but note the relatively weak contribution of target features when a target does not appear in a scene. This was not expected based on existing literature (Zelinsky et al., 2013) and suggests that object-recognition uncertainty or bottom–up saliency may exert stronger attention control on TA search. We will further elaborate on these observations in separate sections devoted to the free- viewing, TP, and TA search tasks.

Although several methods are available to conduct more quantitative analyses, we focused on just two for characterizing the importance of our factors in predicting attention: generalized linear mixed models (GLMMs) and normalized scanpath saliency (NSS). We use GLMM as our main method of quantifying the unique contribution of the different priority maps in the selection of image locations (patches) for fixation. GLMMs extend linear mixed models by allowing response variables from different distributions (e.g., binary responses), thereby enabling models to incorporate both fixed effects (e.g., priority maps) and random effects (e.g., subjects, image instances, target objects) into the prediction of a linear response variable (here, the selection or non-selection of an image patch for fixation). For this reason, we believe GLMM to be the analytical tool best suited to our goal of better understanding the factors contributing to attention prediction. We used the Statistics and Machine Learning Toolbox from MATLAB R2020a (MathWorks, Natick, MA) for our GLMM implementation. In the Supplementary Material, we also report a parallel set of analyses using NSS rather than GLMM, for readers who may be more familiar with that metric. The two analyses yielded highly similar patterns, and our main conclusions do not change depending on our use of one analytical tool or the other.

**Figure 3.**
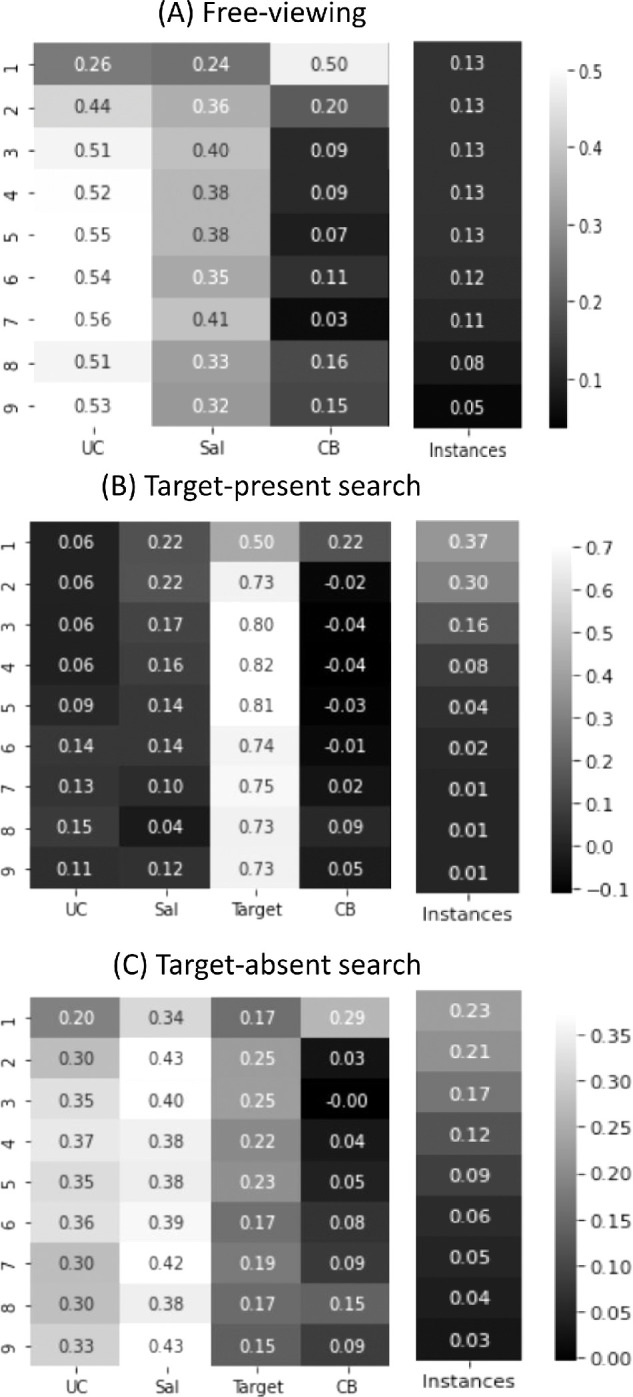
Model predictions for the first nine new fixations showing the relative importance (*z*-statistic of the priority map in a GLMM analysis) of object recognition uncertainty, bottom–up saliency, target features, and center bias in free viewing (A), TP search (B), and TA search (C). Brightness codes greater contribution. Instances show the proportion of images contributing to each fixation prediction. Note that instances sum to 1 over the column and that the factor weights sum to 1 over each row.

Following Hayes and Henderson (2019), as a pre-processing step we first histogram-matched the priority maps to the ground-truth FDMs in order to make the distributions of intensities on these maps more comparable. Following Nuthmann and Einhaüser (2015), we then divided each image into an 8 × 6 grid (yielding 48 scene patches) for our GLMM analyses. For each image patch, we obtained the average priority map value over the corresponding region on each of the feature maps, and for each observer and image we coded whether that patch was fixated (1) or not (0). The priority maps were normalized within a range of 0 to 1 by min–max normalization. The GLMM observation matrix therefore was comprised of *n_image_* × *n_subjects_* × 48 entries of zeros and ones, where *n_image_* and *n_subjects_* are the number of image instances in the dataset and the number of subjects viewing each image. Finally, given that the data are binary, we conducted a logit transformation before modeling the probabilities. For both the free-viewing and search tasks, we included scene type as a random variable. Additionally, our models of TP and TA search include object type as a random variable so as to capture variance attributable to the search target. Figure 3 reports normalized *z*-statistics for GLMMs built for the different feature priority maps. This statistic was shown to vary proportionally to the success of a priority map in predicting fixations in the images, enabling a direct relative comparison between features (Nuthmann & Einhaüser, 2015). Table 1 reports the actual *z* values and standard errors (SEs), as well as *p* values indicating the significance of the unique contribution of a feature to the prediction.

**Table 1.** Standard errors, *z* values, and *p* values for significance tests conducted on the different priority maps from our GLMM analysis.

Free-viewing				
Fixation	Uncertainty	Saliency		Center Bias
1	*SE* = 0*.*151, *z* = 16*.*77, *p <* 0*.*01	*SE* = 0*.*171, *z* = 15*.*61, *p <* 0*.*01		*SE* = 0*.*142, *z* = 32*.*31, *p <* 0*.*01
2	*SE* = 0*.*151, *z* = 21*.*33, *p <* 0*.*01	*SE* = 0*.*166, *z* = 17*.*63, *p <* 0*.*01		*SE* = 0*.*152, *z* = 9*.*48, *p <* 0*.*01
3	*SE* = 0*.*154, *z* = 20*.*65, *p <* 0*.*01	*SE* = 0*.*169, *z* = 16*.*29, *p <* 0*.*01		*SE* = 0*.*161, *z* = 3*.*86, *p <* 0*.*01
4	*SE* = 0*.*155, *z* = 20*.*30, *p <* 0*.*01	*SE* = 0*.*172, *z* = 14*.*74, *p <* 0*.*01		*SE* = 0*.*163, *z* = 3*.*67, *p <* 0*.*01
5	*SE* = 0*.*157, *z* = 20*.*60, *p <* 0*.*01	*SE* = 0*.*175, *z* = 14*.*28, *p <* 0*.*01		*SE* = 0*.*167, *z* = 2*.*63, *p <* 0*.*01
6	*SE* = 0*.*161, *z* = 19*.*57, *p <* 0*.*01	*SE* = 0*.*181, *z* = 12*.*73, *p <* 0*.*01		*SE* = 0*.*169, *z* = 4*.*00, *p <* 0*.*01
7	*SE* = 0*.*171, *z* = 18*.*75, *p <* 0*.*01	*SE* = 0*.*190, *z* = 13*.*55, *p <* 0*.*01		*SE* = 0*.*185, *z* = 1*.*14, *p* = 0*.*25
8	*SE* = 0*.*198, *z* = 14*.*35, *p <* 0*.*01	*SE* = 0*.*222, *z* = 9*.*41, *p <* 0*.*01		*SE* = 0*.*203, *z* = 4*.*58, *p <* 0*.*01
9	*SE* = 0*.*257, *z* = 10*.*90, *p <* 0*.*01	*SE* = 0*.*289, *z* = 6*.*57, *p <* 0*.*01		*SE* = 0*.*264, *z* = 3*.*15, *p <* 0*.*01
Target-present search				
				
Fixation	Uncertainty	Saliency	Target	Center Bias

1	*SE* = 0*.*161, *z* = 18*.*88, *p <* 0*.*01	*SE* = 0*.*130, *z* = 64*.*27, *p <* 0*.*01	*SE* = 0*.*097, *z* = 148*.*22, *p <* 0*.*01	*SE* = 0*.*161, *z* = 66*.*19, *p <* 0*.*01
2	*SE* = 0*.*192, *z* = 15*.*47, *p <* 0*.*01	*SE* = 0*.*157, *z* = 55*.*39, *p <* 0*.*01	*SE* = 0*.*103, *z* = 180*.*76, *p <* 0*.*01	*SE* = 0*.*288, *z* = *−*4*.*18, *p <* 0*.*01
3	*SE* = 0*.*263, *z* = 10*.*28, *p <* 0*.*01	*SE* = 0*.*232, *z* = 28*.*92, *p <* 0*.*01	*SE* = 0*.*139, *z* = 134*.*26, *p <* 0*.*01	*SE* = 0*.*410, *z* = *−*6*.*18, *p <* 0*.*01
4	*SE* = 0*.*381, *z* = 6*.*57, *p <* 0*.*01	*SE* = 0*.*345, *z* = 16*.*50, *p <* 0*.*01	*SE* = 0*.*201, *z* = 87*.*36, *p <* 0*.*01	*SE* = 0*.*572, *z* = *−*4*.*50, *p <* 0*.*01
5	*SE* = 0*.*509, *z* = 6*.*02, *p <* 0*.*01	*SE* = 0*.*491, *z* = 9*.*44, *p <* 0*.*01	*SE* = 0*.*282, *z* = 56*.*47, *p <* 0*.*01	*SE* = 0*.*731, *z* = *−*2*.*09, *p* = 0*.*036
6	*SE* = 0*.*608, *z* = 6*.*82, *p <* 0*.*01	*SE* = 0*.*626, *z* = 6*.*80, *p <* 0*.*01	*SE* = 0*.*374, *z* = 37*.*10, *p <* 0*.*01	*SE* = 0*.*866, *z* = *−*0*.*50, *p* = 0*.*612
7	*SE* = 0*.*766, *z* = 4*.*74, *p <* 0*.*01	*SE* = 0*.*824, *z* = 3*.*74, *p <* 0*.*01	*SE* = 0*.*473, *z* = 28*.*20, *p <* 0*.*01	*SE* = 1*.*023, *z* = 0*.*91, *p* = 0*.*357
8	*SE* = 0*.*894, *z* = 4*.*61, *p <* 0*.*01	*SE* = 1*.*070, *z* = 1*.*25, *p* = 0*.*210	*SE* = 0*.*586, *z* = 23*.*07, *p <* 0*.*01	*SE* = 1*.*147, *z* = 2*.*78, *p* = 0*.*005
9	*SE* = 1*.*150, *z* = 2*.*68, *p* = 0*.*007	*SE* = 1*.*126, *z* = 2*.*92, *p* = 0*.*003	*SE* = 0*.*690, *z* = 18*.*64, *p <* 0*.*01	*SE* = 1*.*392, *z* = 1*.*17, *p* = 0*.*238
Target-absent search				
				
Fixation	Uncertainty	Saliency	Target	Center Bias
				
1	*SE* = 0*.*047, *z* = 46*.*15, *p <* 0*.*01	*SE* = 0*.*044, *z* = 78*.*05, *p <* 0*.*01	*SE* = 0*.*060, *z* = 39*.*53, *p <* 0*.*01	*SE* = 0*.*042, *z* = 65*.*85, *p <* 0*.*01
2	*SE* = 0*.*049, *z* = 52*.*12, *p <* 0*.*01	*SE* = 0*.*048, *z* = 74*.*95, *p <* 0*.*01	*SE* = 0*.*061, *z* = 43*.*10, *p <* 0*.*01	*SE* = 0*.*050, *z* = 4*.*96, *p <* 0*.*01
3	*SE* = 0*.*056, *z* = 45*.*72, *p <* 0*.*01	*SE* = 0*.*057, *z* = 51*.*96, *p <* 0*.*01	*SE* = 0*.*072, *z* = 31*.*97, *p <* 0*.*01	*SE* = 0*.*059, *z* = *−*0*.*463, *p* = 0*.*64
4	*SE* = 0*.*067, *z* = 37*.*09, *p <* 0*.*01	*SE* = 0*.*069, *z* = 38*.*48, *p <* 0*.*01	*SE* = 0*.*090, *z* = 21*.*760, *p <* 0*.*01	*SE* = 0*.*068, *z* = 3*.*59, *p <* 0*.*01
5	*SE* = 0*.*080, *z* = 28*.*41, *p <* 0*.*01	*SE* = 0*.*082, *z* = 30*.*78, *p <* 0*.*01	*SE* = 0*.*108, *z* = 18*.*45, *p <* 0*.*01	*SE* = 0*.*08, *z* = 4*.*08, *p <* 0*.*01
6	*SE* = 0*.*093, *z* = 23*.*50, *p <* 0*.*01	*SE* = 0*.*095, *z* = 25*.*82, *p <* 0*.*01	*SE* = 0*.*136, *z* = 11*.*50, *p <* 0*.*01	*SE* = 0*.*093, *z* = 5*.*17, *p <* 0*.*01
7	*SE* = 0*.*111, *z* = 16*.*90, *p <* 0*.*01	*SE* = 0*.*109, *z* = 23*.*89, *p <* 0*.*01	*SE* = 0*.*161, *z* = 10*.*84, *p <* 0*.*01	*SE* = 0*.*107, *z* = 4*.*96, *p <* 0*.*01
8	*SE* = 0*.*127, *z* = 14*.*23, *p <* 0*.*01	*SE* = 0*.*128, *z* = 17*.*80, *p <* 0*.*01	*SE* = 0*.*194, *z* = 7*.*94, *p <* 0*.*01	*SE* = 0*.*121, *z* = 7*.*196, *p <* 0*.*01
9	SE = 0.144, *z* = 13.18, *p* < 0.01	SE = 0.144, *z* = 17.14, *p* < 0.01	SE = 0.228, *z* = 5.87, *p* < 0.01	SE = 0.140, *z* = 3.76, *p* < 0.01

### Free viewing

Figure 3A shows normalized GLMM *z*-statistics indicating the unique contribution of priority map feature in predicting FDMs computed for each of the first nine new fixations made during free viewing. The rightmost column shows the relative number of image “instances” over which the GLMM *z*-statistics are estimated. Brightness codes a greater contribution of a feature bias, or more instances. The nine rows correspond to the first nine new fixations made during the free-viewing task, with the weightings of values for each row normalized to sum to 1. For example, the most weighted feature for predicting attention was object recognition uncertainty on fixation 7. Note that instances are also normalized to sum to 1, only this normalization is column-wise. Values from top to bottom therefore indicate the proportion of images having exactly one new fixation, exactly two new fixations, etc.

The clear pattern from these data is that uncertainty was better than both saliency and center bias in predicting free-viewing fixations, and that this was broadly true regardless of the order of the fixation in the scanpath. The only exception to this pattern was in the first fixation, where center bias was the best predictor of attention. Feature weights were significant for all fixations, except for center bias on fixation 7, as indicated by an independent *t*-test analysis of our GLMM model (Table 1, top). Note that these and all subsequent statistical comparisons were Bonferroni corrected to avoid inflated type I errors by multiple testing.

### Target-present search

Figure 3B shows the corresponding analyses for a search task where an exemplar of the target category appeared in the search scene. The most salient pattern in these data is the superiority of target features in predicting overt search behavior (Table 1, middle), which was clear by even the first saccade and peaked over fixations three to five. The slight decline in target-feature weighting observed over fixations six to nine should be interpreted with caution because the mean number of fixations required to find the target in the COCO-Search18 dataset was 1.85, meaning that there were relatively few trials having five or more fixations. Moreover, these trials likely represent the more difficult searches where the target guidance signal might be smaller. The dominance of a target bias in our weighting came largely at the expense of a diminished center bias, perhaps due in part to target objects being prevented from appearing at center locations in COCO-Search18. Target-feature dominance also diminished the unique contributions of uncertainty and saliency, rendering both small with saliency being narrowly better.

### Target-absent search

Figure 3C shows these analyses again for a search task, except this time for trials when a target exemplar did not appear in the search image. A notable pattern here is the stark reversal found in the importance of the target features. Whereas for TP search this factor easily dominated the others throughout the scanpath, in TA search this factor was weighted among the lowest over all the scanpath fixations. This greatly diminished role of target features corresponded to an increased role for recognition uncertainty and saliency, with both factors uniformly predicting TA search fixations better than target features. Saliency and uncertainty were more closely weighted, although saliency was generally more predictive of fixations during TA search.

### Alternative definitions of object recognition uncertainty

We have shown that a model derived from object recognition uncertainty better predicts free-viewing fixations than a bottom–up model of saliency. Still not clear, however, is whether this better prediction is due to the specific formulation of object recognition uncertainty used in that model or because it is more simply object based and uses information about objects to predict attention. Our model comparison focuses on this distinction. Note that we do not compare our method to deep-network models of “saliency” because these models are trained on fixation behavior and are therefore not strictly object based. Objects are just one of many factors contributing to fixation selection (with, for example, bottom–up saliency and center bias being others), and this uninterpretable mixture of factors introduces confounds with respect to our goal of estimating the contributions of specific factors. Relatedly, because these models are trained explicitly on fixation behavior to predict fixation behavior, they *should* be more predictive than a model built exclusively on object uncertainty. But, again, confirming this expectation would not advance our goal of identifying factors contributing to visual behavior. What we do instead is to compare the free-viewing fixation behavior predicted by the object recognition uncertainty model to two other models that are comparable in architecture but use different definitions of object-based uncertainty to prioritize attention.

### Object label entropy

Object uncertainty is a topic that has been studied in computer vision, where methods commonly characterize uncertainty in the detection (Jiang, Luo, Mao, Xiao, & Jiang, 2018; He, Zhu, Wang, Savvides, & Zhang, 2019; Wang et al., 2020) and recognition (Miller, Nicholson, Dayoub, & Sünderhauf, 2018; Hall et al., 2020; Meyer & Thakurdesai, 2020) of objects in images. However, these approaches define object uncertainty very differently than how we defined it in our study. Object detection uncertainty refers to the uncertainty in the location of a detected object, meaning the preciseness of its segmentation or the location of its detection bounding box. Object recognition uncertainty refers to a confidence in the classification label of a detected object, rather than our conceptualization of recognition uncertainty in terms of the number of objects competing for a given object proposal. A very recent probabilistic object recognition approach (Hall et al., 2020) quantified both spatial (detection) and semantic (recognition) uncertainties of the detections, but more related to our work is that of Miller et al. (2018), who used the entropy of the object label (category) probabilities as a measure of the uncertainty in object recognition.

Building on the model from Miller et al. (2018) and inspired by information theory (Bruce & Tsotsos, 2009), here we compute MaskRCNN object confidence scores for proposal bounding boxes and use the entropy in these scores as a measure of uncertainty. Greater entropy in these scores for a given bounding box would indicate more competition among object classes for this box and, therefore, greater uncertainty. Uncertainty scores for all overlapping boxes are summed to obtain the final uncertainty map. This method can be formalized as

(3) $$\begin{array}{c} UC_{E}=\sum_{b=1}^{B}G_{B_{center}}^{\sigma }\left(-\sum_{p=1}^{f_{b}}w_{p}\;log\;w_{p}\right)\; \end{array}$$

where *B* is the number of object proposal bounding boxes in image *I*, and *f_b_* is the number of objects competing for object proposal *b*, having confidence *w_p_* ≥ *Th_conf._*. $\;G_{B_{center}}^{\sigma }$ is a Gaussian kernel with *G* centered at the proposal box center (*B_center_*) with *SD σ*.

### Pixel-wise uncertainty

The object recognition model from our GLMM analyses defined recognition uncertainty in terms of objects competing for an object proposal in a visual input. The second alternative measure of uncertainty that we consider is related but measures uncertainty at the pixel level. For a given pixel *P*, a *pixel-wise uncertainty* measure determines the number of object categories seeking to claim it, with the goal being the classification of every pixel with a category label. The premise is that the degree of uncertainty in how a pixel should be assigned to an object class increases with the number of object bounding boxes seeking to claim that pixel as part of a different object representation. Using the same MaskRCNN method and parameter settings reported in the Methods section, we implemented this pixel-wise uncertainty metric by simply incrementing by 1 an uncertainty value obtained for pixel *P* for every bounding box (*B_I_*) that encloses *P*. An uncertainty map is thus obtained by computing an uncertainty value for each pixel reflecting an unweighted sum of overlapping bounding boxes enclosing a given pixel. This method can be formalized as

(4) $$\begin{array}{c} UC_{P}\left(x,y\right)=G_{im}^{\sigma }\sum_{p=1}^{f_{b}}F_{p}\left(x,y\right)\; \end{array}$$

where *F_p_*(*x, y*) = 1 if the bounding box *p* encloses the point *P*(*x, y*), and $\;G_{im}^{\sigma }$ is a Gaussian kernel (size = one-fourth of the image height, *h_im_*) applied on the resulting image for smoothing. The greater the number of object bounding boxes enclosing a pixel, the greater the uncertainty attributed to a pixel enclosed by the boxes.

### Model comparison

**Figure 4.**
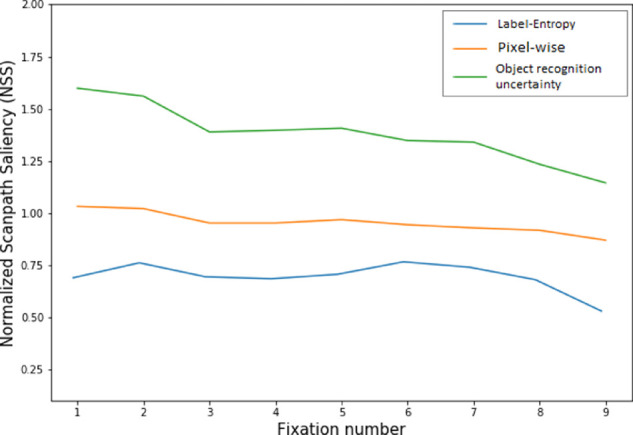
NSS prediction accuracy as a function of fixation number for the label-entropy, pixel-wise, and object recognition uncertainty models.

Figure 4 shows fixation-by-fixation predictions from the two alternative uncertainty models (label-entropy and pixel-wise) and the originally formulated object recognition uncertainty model. To evaluate how well the priority maps from each model could predict the ground-truth FDMs, we used the NSS metric. NSS is computed for a given image by taking the average of the model predictions at each of the fixation locations, where the model predictions were first normalized to have zero mean and unit standard deviation. Thus, when NSS = 1, the locations of the ground-truth fixations are being predicted by a model 1 *SD* above average, and when NSS = 0 a model is predicting fixation locations no better than chance. NSS penalizes false positives and false negatives symmetrically, and as noted in Bylinskii, Judd, Oliva, Torralba, and Durand (2018), is a discrete approximation of a simple Pearson's correlation between the priority map of a model and an observed fixation-density map. We chose to use NSS rather than the correlation method because NSS is parameter free (the sigma of the Gaussian used to create the FDM can be treated as a fit parameter), although the metrics produce similar results (Li, Xia, Song, Fang, & Chen, 2015).

**Figure 5.**
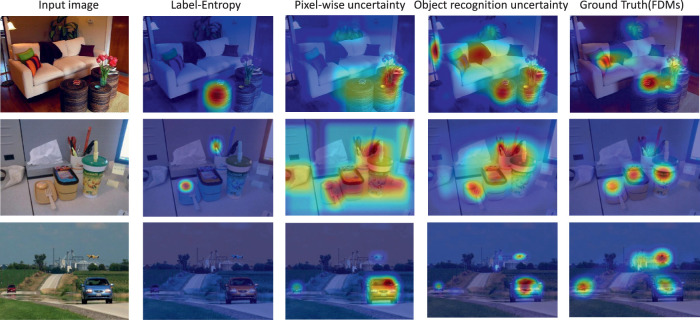
Predictions from three object recognition uncertainty models (middle three columns) and ground-truth fixation-density maps (right) superimposed over three representative images (left).

Using this metric, and averaging NSS scores over the first nine new fixations, we obtained scores of 0.74, 0.91, and 1.35 for the label-entropy, pixel-wise uncertainty and for the originally formulated object recognition uncertainty models, respectively. This advantage for the object recognition uncertainty model existed for each of the first nine new fixations. Figure 5 supports this finding with a visualization of model predictions for representative images from the OSIE dataset. Consistent with the NSS analysis, predictions from our original object recognition uncertainty model appear the best match to ground-truth free-viewing fixations, suggesting that this metric may be the most psychologically meaningful of the three considered here.

## Discussion and future directions

Our goal in this study was to better understand the relative importance of different factors (namely, center bias, target bias, bottom–up saliency, and object recognition uncertainty) in predicting gaze fixations during free-viewing and search tasks. We report several patterns, some expected and some unexpected. One expected pattern was that center bias played a strong role in predicting the first new fixations in free-viewing and TA search tasks compared with the importance of this factor throughout the remainder of the scanpath. We interpret this pattern as suggesting that, in the absence of a strong target guidance signal attracting attention to a location in the visual periphery, people make relatively small amplitude initial saccades away from the starting center location (Zelinsky, 2012), thus creating the behavioral data pattern captured by the center-bias model. Also as expected, we found that target features introduced a strong bias on attracting attention during TP categorical search (Malcolm & Henderson, 2009; Schmidt & Zelinsky, 2009; Yang & Zelinsky, 2009). This target bias appeared in the very first fixation and dominated the allocation of attention throughout the nine-fixation scanpath. Unexpectedly, however, the target bias did not extend to TA search, where target features ranked among the poorest in predicting attention. Based on previous work showing that the target guidance signal was sufficiently strong on TA trials to decode the target category (Zelinsky, Peng, & Samaras, 2013), we expected to find some role for target guidance. However, that study used only four-object search arrays and purposefully inserted non-target objects that were rated as visually similar to the target category. Our findings suggest that target guidance, compared with other factors, is relatively weak in the case of TA categorical search and perhaps only meaningful when the search context can be significantly constrained.

Another unexpected finding from our study was the strong role played by object recognition uncertainty in controlling gaze during free viewing, given that this factor has been relatively neglected in the study of attention control. For free viewing, uncertainty played a greater role than saliency in guiding overt attention. We also found saliency to be a significant factor affecting attention, just to a lesser degree than uncertainty. Saliency gave uniformly better pre dictions than center bias regardless of task, except for the first new fixation during free viewing where center bias was most predictive. Saliency was even more predictive than target features in the case of TA search. TA search therefore appears to be more exploratory than guided by target features, resulting in larger roles played by image saliency and object uncertainty. Comparing uncertainty to saliency during search, we found saliency to be the clearly better predictor over the entire TA search scanpath, with this advantage extending to TP search, although becoming smaller. We therefore conclude that, whereas bottom–up saliency is generally more predictive in the case of visual search, object recognition uncertainty is better than bottom–up saliency in predicting fixations made during free viewing. Ironically, given that saliency models were developed in the context of free-viewing tasks, uncertainty in object recognition dominated saliency as a factor biasing the first nine new fixations.

We propose that a basic factor affecting the allocation of visual attention is a need to recognize objects. It is through object recognition that a meaningful label becomes attached to previously unlabeled visual input, thereby enabling all further complex motor and cognitive interactions with the object. It therefore stands to reason that an object in an image having more than one label will create a recognition-based dissonance that attracts attention in an effort to resolve the object recognition uncertainty. It is unclear how our formulation of object recognition uncertainty relates to *meaning maps* (Henderson & Hayes, 2017), and future work will explore whether there is more to meaning than just object recognition in attention control. What we can say, however, is that our formulation of object-based control uses computer vision methods that rely solely on pixels and can therefore be applied to a limitless number of images, making it preferable in this sense to the hand-labeling method used to create meaning maps.

## Supplementary Material

Supplement 1
