# Supplementary material for: Weighting the factors affecting attention guidance during free viewing and visual search: The unexpected role of object recognition uncertainty
Source: J Vis. 2022 Mar 24;22(4):13. doi: 10.1167/jov.22.4.13 (PMC8963662; doi:10.1167/jov.22.4.13)
Supplement: Supplement 1 [file jovi-22-4-13_s001.pdf]

Supplementary

Analyses using the Normalized Scanpath Saliency metric

To complement the GLMM analyses reported in the main text, here we report parallel analyses that we conducted of the fixation-by-fixation attention behavior using the Normalized Scanpath Saliency (NSS) metric. NSS is computed for a given image by taking the average of the model predictions at each of the fixation locations, where the model predictions were first normalized to have zero mean and unit standard deviation. We implemented NSS using the *Pysaliency* (Kümmerer, 2021) toolbox in Python. Figure S1 shows NSS scores indicating how well each of the tested models predicted FDMs computed after each of the first nine new fixations made during the free viewing and visual search tasks, plotted with the relative number of image ”instances” over which the NSS scores are averaged. This figure is therefore an NSS version of Figure 3 in the main text, where NSS scores replace the z-statistics from the GLMM analyses.

Comparing the two figures, we see very similar patterns in the relative contributions of the factors. Table S1 provides the results of statistical tests performed between pairs of factors using the NSS scores. Finding such largely converging patterns from two very different analyses is reassuring. As in the case of the GLMM analyses, our NSS analysis of the free-viewing data found center bias to be the best predictor of the first new fixation but that object recognition uncertainty dominated both saliency and center bias for the remainder of the free-viewing scanpath. We also found similar patterns for target-present search. As in the GLMM analysis, the NSS analysis showed that target features dominated predictions over the entire search scanpath. Also similar to the GLMM analysis is that saliency is shown to be more predictive than uncertainty in the early search fixations. For target-absent search, the GLMM and NSS analyses differed slightly. Whereas GLMM suggested a clear predictive advantage for saliency over uncertainty, the NSS analysis did not find this difference to be significant over fixations 3-7 (Bonferroni corrected). Compared to GLMM, NSS therefore slightly overstates the role of object uncertainty relative to saliency throughout the middle of the target-absent search scanpath.

Free-viewing

Fixation	Uncertainty-Saliency	Uncertainty-Center Bias	Saliency-Center Bias
1	$t = 3.76, p < 0.001$	$t = -2.37, p < 0.02$	$t = -6.73, p < 0.001$
2	$t = 4.50, p < 0.001$	$t = 7.68, p < 0.001$	$t = 2.75, p < 0.01$
3	$t = 6.11, p < 0.001$	$t = 7.64, p < 0.001$	$t = 1.61, p = 0.10$
4	$t = 6.35, p < 0.001$	$t = 8.96, p < 0.001$	$t = 2.52, p < 0.02$
5	$t = 5.30, p < 0.001$	$t = 9.14, p < 0.001$	$t = 3.37, p < 0.001$
6	$t = 6.72, p < 0.001$	$t = 8.30, p < 0.001$	$t = 1.75, p = 0.08$
7	$t = 6.30, p < 0.001$	$t = 9.24, p < 0.001$	$t = 3.18, p < 0.01$
8	$t = 4.38, p < 0.001$	$t = 6.37, p < 0.001$	$t = 2.07, p < 0.05$
9	$t = 4.53, p < 0.001$	$t = 4.19, p < 0.001$	$t = 0.08, p = 0.93$

Target-present search

Fix.	Uncertainty-Saliency	Uncertainty-Target	Uncertainty-Center Bias	Saliency-Target	Saliency-Center Bias	Target-Center Bias
1	$t = -11.07, p < 0.001$	$t = -31.51, p < 0.001$	$t = -8.32, p > 0.4$	$t = -23.93, p < 0.001$	$t = 5.79, p < 0.001$	$t = 28.94, p < 0.001$
2	$t = -6.46, p < 0.001$	$t = -50.50, p < 0.001$	$t = 19.40, p < 0.001$	$t = -43.88, p < 0.001$	$t = 19.51, p < 0.001$	$t = 60.34, p < 0.001$
3	$t = -4.78, p < 0.001$	$t = -41.83, p < 0.001$	$t = 13.85, p < 0.001$	$t = -38.63, p < 0.001$	$t = 15.54, p < 0.001$	$t = 49.18, p < 0.001$
4	$t = -2.66, p < 0.01$	$t = -29.64, p < 0.001$	$t = 10.72, p < 0.001$	$t = -27.23, p < 0.001$	$t = 11.50, p < 0.001$	$t = 34.98, p < 0.001$
5	$t = 0.16, p = 0.86$	$t = -20.44, p < 0.001$	$t = 8.23, p < 0.001$	$t = -21.27, p < 0.001$	$t = 8.63, p < 0.001$	$t = 25.29, p < 0.001$
6	$t = 2.45, p < 0.02$	$t = -13.23, p < 0.001$	$t = 6.56, p < 0.001$	$t = -14.94, p < 0.001$	$t = 4.85, p < 0.001$	$t = 17.15, p < 0.001$
7	$t = -0.19, p = 0.84$	$t = -9.66, p < 0.001$	$t = 3.53, p < 0.001$	$t = -9.58, p < 0.001$	$t = 3.42, p < 0.001$	$t = 11.89, p < 0.001$
8	$t = 1.72, p = 0.08$	$t = -6.82, p < 0.001$	$t = 4.01, p < 0.001$	$t = -8.52, p < 0.001$	$t = 3.15, p < 0.01$	$t = 10.00, p < 0.001$
9	$t = 0.35, p = 0.72$	$t = -6.59, p < 0.001$	$t = 2.89, p < 0.01$	$t = -6.94, p < 0.001$	$t = 2.89, p < 0.01$	$t = 8.21, p < 0.001$

Target-absent search

Fix.	Uncertainty-Saliency	Uncertainty-Target	Uncertainty-Center Bias	Saliency-Target	Saliency-Center Bias	Target-Center Bias
1	$t = -15.60, p < 0.001$	$t = 2.76, p < 0.01$	$t = -17.97, p < 0.001$	$t = 11.61, p < 0.001$	$t = -1.37, p = 0.17$	$t = -13.90, p < 0.001$
2	$t = -3.81, p < 0.001$	$t = -2.49, p < 0.02$	$t = 11.72, p < 0.001$	$t = -1.35, p = 0.17$	$t = 15.41, p < 0.001$	$t = 10.31, p < 0.001$
3	$t = 1.69, p = 0.09$	$t = -1.92, p = 0.05$	$t = 14.46, p < 0.001$	$t = -4.03, p < 0.001$	$t = 13.06, p < 0.001$	$t = 12.11, p < 0.001$
4	$t = 1.55, p = 0.12$	$t = 0.19, p = 0.84$	$t = 10.06, p < 0.001$	$t = -2.59, p < 0.01$	$t = 8.62, p < 0.001$	$t = 7.88, p < 0.001$
5	$t = 1.59, p = 0.11$	$t = -1.20, p = 0.22$	$t = 8.52p < 0.001$	$t = -3.48, p < 0.001$	$t = 6.96, p < 0.001$	$t = 7.96, p > 0.7$
6	$t = -0.72, p = 0.47$	$t = 1.53, p = 0.12$	$t = 5.20, p < 0.001$	$t = 1.15, p = 0.24$	$t = 5.85, p < 0.001$	$t = 2.46, p < 0.02$
7	$t = -1.55, p = 0.12$	$t = -0.41, p = 0.67$	$t = 3.89, p < 0.001$	$t = 0.02, p = 0.98$	$t = 5.51, p < 0.001$	$t = 3.46, p < 0.01$
8	$t = -2.32, p < 0.02$	$t = 1.27, p = 0.20$	$t = 2.89, p < 0.01$	$t = 1.94, p = 0.05$	$t = 5.33, p < 0.001$	$t = 1.62, p = 0.10$
9	$t = -2.90, p < 0.01$	$t = 0.52, p = 0.59$	$t = 2.24, p < 0.03$	$t = 1.03, p = 0.30$	$t = 5.17, p < 0.001$	$t = 1.42, p = 0.15$

Table S1: Student's t-test results on NSS scores, Bonferroni corrected, for pairwise comparisons between models.

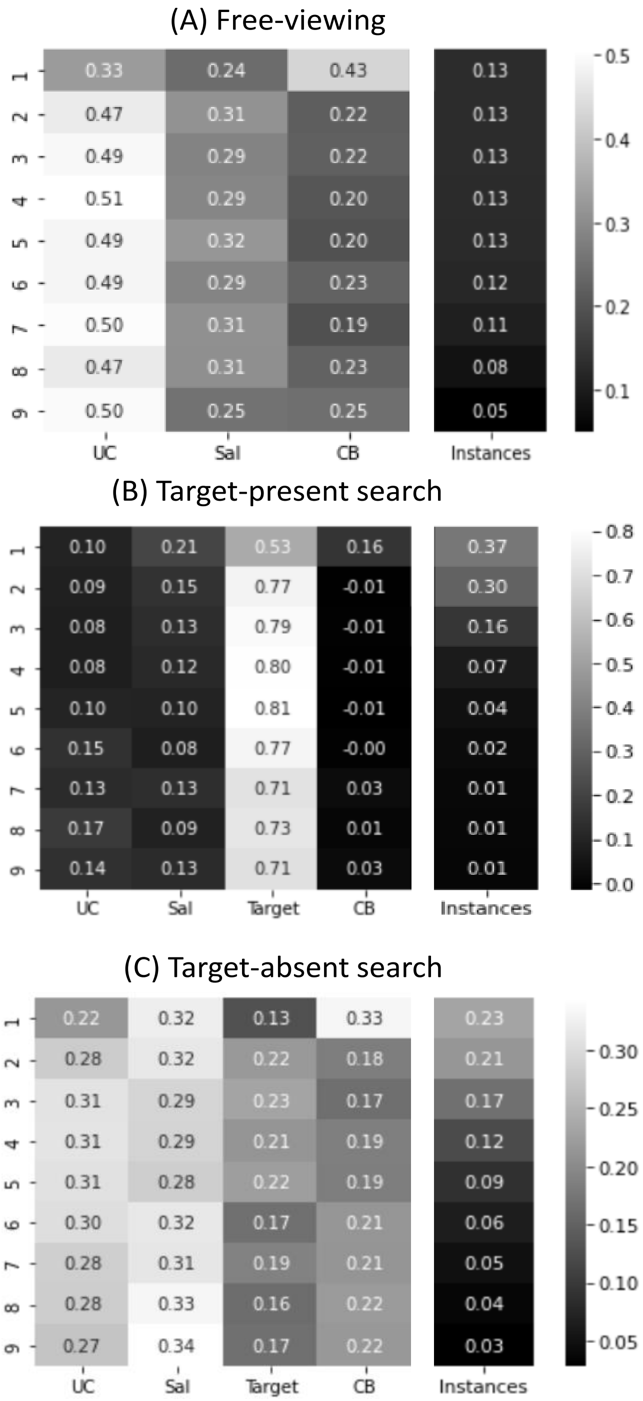

Figure S1: Model predictions for the first nine new fixation showing the relative importance (NSS scores normalized column-wise) of object recognition uncertainty, bottom-up saliency, target features and center bias in free-viewing (A), target-present search (B), and target-absent search (C). Brightness codes greater contribution. Instances show the proportion of images contributing to each fixation prediction. Note that instances sum to 1 over the column, and that the factor weights sum to 1 over each row.
